# The Impact of *LEP* G-2548A and *LEPR* Gln223Arg Polymorphisms on Adiposity, Leptin, and Leptin-Receptor Serum Levels in a Mexican Mestizo Population

**DOI:** 10.1155/2015/539408

**Published:** 2015-05-10

**Authors:** Efraín Chavarria-Avila, Mónica Vázquez-Del Mercado, Eduardo Gomez-Bañuelos, Sandra-Luz Ruiz-Quezada, Jorge Castro-Albarran, Lizeth Sánchez-López, Beatriz Teresita Martín-Marquez, Rosa-Elena Navarro-Hernández

**Affiliations:** ^1^Centro Universitario de Ciencias de la Salud, Universidad de Guadalajara, Sierra Mojada No. 950, Colonia Independencia, 44340 Guadalajara, JAL, Mexico; ^2^Departamento de Farmacobiología, Centro Universitario de Ciencias Exactas e Ingenierías, Universidad de Guadalajara, Boulevard Marcelino García Barragán No. 1421, Esquina Calzada Olímpica, 44430 Guadalajara, JAL, Mexico; ^3^UDG-CA-701, Grupo de Investigación Inmunometabolismo en Enfermedades Emergentes (GIIEE), Centro Universitario de Ciencias de la Salud, Universidad de Guadalajara, Sierra Mojada No. 950, Colonia Independencia, 44340 Guadalajara, JAL, Mexico; ^4^Instituto de Investigación en Reumatología y del Sistema Musculo Esquelético, Centro Universitario de Ciencias de la Salud, Universidad de Guadalajara, Sierra Mojada No. 950, Colonia Independencia, 44340 Guadalajara, JAL, Mexico; ^5^Servicio de Reumatología, División de Medicina Interna, Hospital Civil “Dr. Juan I. Menchaca”, Universidad de Guadalajara, Salvador de Quevedo y Zubieta No. 750, 44340 Guadalajara, JAL, Mexico; ^6^HMIELM, Secretaria de Salud Jalisco, Avenida Constituyentes 1075, Colonia Moderna, 44190 Guadalajara, JAL, Mexico; ^7^Instituto Tecnológico y de Estudios Superiores de Monterrey (ITESM), Campus Guadalajara, Avenida General Ramón Corona 2514, San Juan de Ocotán, 45201 Zapopan, JAL, Mexico

## Abstract

The polymorphisms in leptin (*LEP* G-2548A) and leptin-receptor (*LEPR* Gln223Arg) seem to influence obesity and lipid metabolism among others. The aim of this study was to investigate the effect of these polymorphisms on adiposity, leptin (sLeptin), and leptin-receptor (sLeptin-receptor) serum concentrations as well as inflammation markers. We included 382 adults originally from Western Mexico. They were genotyped by PCR-RFLP. Obese individuals showed higher sLeptin (58.2 ± 31.35 ng/mL) but lower sLeptin-receptor (12.6 ± 3.74 ng/mL) levels than normal weight ones (17.6 ± 14.62 ng/mL, 17.4 ± 4.62 ng/mL, resp.), *P* < 0.001. Obese subjects carriers of Arg/Arg genotype had more (*P* = 0.016) sLeptin-receptor (14.7 ± 4.96 ng/mL) and less (*P* = 0.004) sLeptin (44.0 ± 28.12 ng/mL) levels than Gln/Gln genotype (11.0 ± 2.92 ng/mL, 80.3 ± 33.24 ng/mL, resp.). Body fat mass was lower (*P* from 0.003 to 0.045) for A/A (36.5% ± 6.80) or Arg/Arg (36.8% ± 6.82) genotypes with respect to G/G (41.3% ± 5.52) and G/A (41.6% ± 5.61) or Gln/Gln (43.7% ± 4.74) and Gln/Arg (41.0% ± 5.52) genotypes carriers. Our results suggest that *LEP* -2548A and *LEPR* 223Arg could be genetic markers of less body fat mass accumulation in obese subjects from Western Mexico.

## 1. Introduction

Obesity is the result of a complex interaction between genetic variability, environment, and sedentary lifestyle [1–4]. It is considered one of the most serious public health problems since its prevalence has worldwide greatly increased during the last three decades [5, 6]; for example, in Mexico over the last health survey the prevalence of obesity was 37.5%, a disturbing situation for the national health system [7, 8]. Additionally, obesity is an important risk factor for developing type 2 diabetes mellitus (T2DM) and cardiovascular diseases [9].

The adult individuals are classified with obesity when their body mass index (BMI) is greater than 30 kg/m^2^ [10, 11] and are characterized by a high proportion of body fat mass; also, during development of obesity increase in a protein hormone called leptin occurs secondary to white adipose tissue accumulation [12].

The leptin is an adipokine secreted by white adipose tissue that regulates food intake and energy expenditure; furthermore, it also plays an important role in glucose homeostasis, immunity, and fertility among others [12–15]. Leptin exerts its action through leptin-receptors, which are transmembrane proteins members of the class I cytokine receptor superfamily, and their pathway involves JAK/STAT, PI3K, and MAPK/ERK systems and PKC [13, 15].

The* leptin* (*LEP*) and* leptin-receptor* (*LEPR*) genes have been studied to find gene variants potentially related to the pathophysiology of obesity, T2DM, and their associated complications. For example, a single nucleotide polymorphism (SNP) in* LEP*, consisting in G to A substitution at nucleotide -2548 upstream from ATG start site (*LEP* G-2548A, rs7799039), in locus 7q31.3, has been associated with increased leptin secretion in adipocytes [16–18], while the* LEPR* gene polymorphism, an A to G transition in exon 6 at nucleotide 668 from the start codon (Gln223Arg, rs1137101), in locus 1p31.3, has been associated with decreased BMI, higher leptin levels, and some types of cancers [19, 20].

There are few studies that associate obesity status with* LEP* G-2548A and* LEPR* Gln223Arg polymorphisms, and, even more important, these associations are shown in a racial dependent fashion [18, 21]; that is why it is necessary to determine their influence on obesity in Mexican population groups [22, 23]. In this context, the aim of this study was to investigate whether two common SNPs in the leptin (*LEP* G-2548A) and its receptor (*LEPR* Gln223Arg) genes are related to adiposity, leptin, and leptin-receptor concentrations in Western Mexican Mestizo population.

## 2. Materials and Methods

### 2.1. Subjects' Assessment

In a cross-sectional study we included 382 unrelated adult Mexican Mestizo subjects (originally from Western Mexico, as well as their parents and grandparents) classified by BMI according to World Health Organization (WHO) criteria as follows: normal range (BMI 18.5–24.9 kg/m^2^, *n* = 154), overweight (BMI 25.0–29.9 kg/m^2^, *n* = 146), and obese (BMI ≥ 30 kg/m^2^, *n* = 82); and for some associations, we consider an additional criterion: BMI ≥ 25.0 kg/m^2^.

Before the enrollment, the participants gave their signature in a consent document after they were informed about all the implications of the study. Appropriate ethical and biosecurity conduct was ensured by the Declaration of Helsinki guidelines and the institutional (Universidad de Guadalajara) review boards' committees.

None of the participants presented signs or symptoms of acute or chronic disease, besides obesity, that is, past history of glucose intolerance, medication, and a stable weight for at least three weeks. Individuals with infectious diseases, hypertension, history of cardiovascular disease, malignancy, and renal and metabolic diseases such as T2DM were not included.

### 2.2. Physical Examination and Medical History

All participants completed a questionnaire to gather personal and family medical history. Blood pressure and heart rate were measured 3 times at 3-minute intervals with the subject in the sitting position, and before the first measurement they had a relaxation period of at least 15 minutes.

### 2.3. Anthropometric Measurements

Height was measured using a stadiometer (Seca GmbH & Co. KG., Hamburg, Germany, to the nearest 1.0 mm), while body weight (to the nearest 0.1 kg), BMI, and body composition (including total muscle and body fat mass percentage) were determined using bioelectrical impedance analysis (TANITA Corporation, TBF304, Tokyo, Japan) [24]. Waist and hip circumferences were measured to the nearest 0.1 cm with an anthropometric fiberglass tape (GULICK, accuracy ± 1 mm; North Coast Medical Inc., Gilroy, CA) following the procedures recommended by Durnin [25, 26].

Four measurements of skinfold thickness (biceps, triceps, subscapular, and suprailiac) were obtained from the right side of the body employing a caliper skinfold Harpenden (with a maximum opening of 80 mm, accuracy of ±0.2 mm, and constant pressure of 10 g/mm^2^; Holtain Ltd., Croswell, UK), according to the procedures recommended by the Anthropometric Indicators Measurement Guide [27, 28]. We also calculated the waist/hip ratio as an indicator of preferential accumulation of fat in the abdomen, rather than in the extremities.

### 2.4. Laboratory Techniques and Procedures

After overnight fast of 12 hours in all participants, we obtained venous blood samples that were allowed to clot at room temperature and were subsequently centrifuged at 1500 RCF (Rotanta 460R, Andreas Hettich GmbH & Co. KG., Germany) for ten minutes. The serum was separated and then stored at −70°C until analysis.

Serum levels of leptin (sLeptin, limit of detection of 0.42 ng/mL, ALPCO Diagnostics, 26-G Keewaydin Drive, Salem, NH) and leptin-receptor (sLeptin-receptor, limit of detection of 0.4 ng/mL, Enzo Life Sciences, Inc., New York, NY, USA) were quantified using ELISA, fasting glucose by oxidase/peroxidase method, and high-sensitivity C reactive protein (CRP) with a limit of detection of 0.15 mg/L (Randox Laboratories, 55 Diamond Road, Crumlin, County Antrim, Northern Ireland, UK) which was determined by immunoturbidimetry. We measured erythrocyte sedimentation rate (ESR) by Wintrobe method and white blood cell count (WBC) with the Cell-Dyn 3700 (Abbott Diagnostics, Abbott Park, IL).

### 2.5. Polymorphisms Analysis

To identify genetic polymorphisms, genomic DNA was isolated from total blood using the modified Miller method [29] and stored at −20°C until use for genotyping. The polymorphisms* LEP* G-2548A and* LEPR* Gln223Arg were analyzed by polymerase chain reaction (PCR) based restriction fragment length polymorphism (RFLP) methods. Primers were as follows: forward 5′-TCCCATGAGAACTATTCTTCTTTTG-3′, reverse 5′-ATATGGCTCCCTTTGCCCGACC-3′ for* LEP* gene; forward 5′-ACCTCTGGTTCCCCAAAAAG-3′, reverse 5′-TCATCATTTTAGTGCATAACTTACCC-3′ for* LEPR* gene.

The PCR procedure was performed with a 25 *µ*L reaction mixture (100 ng of DNA, 4 nM of each primer, 2.5 mM of each dNTP, 2.5 mM of MgCl_2_, 0.025 U* Taq* polymerase, and 1x PCR buffer, Invitrogen) and consisted of an initial melting step of 2 min at 94°C, followed by 35 cycles of 30 s at 94°C, 45 s at 60°C (for* LEP*), 45 s at 56°C (for* LEPR*), and 45 s at 72°C, and a final elongation step of 6 min at 72°C.

From the PCR amplified products, 15 *µ*L was digested with restriction enzymes (New England Biolabs^©^ Inc., Ipswich, MA),* Hha*I [20] for* LEP* and* Msp*I [20] for* LEPR*, for two hours at 37°C. Then, the PCR products and the fragments obtained from enzyme digestion were separated on 3% agarose gels stained with ethidium bromide 0.01 *µ*g.

As from agarose gels, it was observed that both polymorphisms exhibited the corresponding single-band pattern of each allele: the* LEP* allele A that lacks* Hha*I restriction site was defined by a 451 bp fragment, while allele G, which contains this restriction site, was characterized by two digested bands of 402 and 49 bp. On the other hand,* LEPR* allele Gln, which is deficient in* Msp*I restriction site, was represented by a 212 bp fragment, whereas allele Arg, containing the restriction site, was represented by two bands of 151 and 61 bp in length. To ensure the accuracy of genotype data, a blank and samples previously confirmed as positive for each genotype were used as controls. All samples were repeated at random to verify the reproducibility.

### 2.6. Statistical Analysis

Data was analyzed with the PASW Statistics program (v18.0; SPSS Inc., Chicago, IL, USA). Results are given as mean ± SD or percentages. The clinical and laboratory characteristics of the study group were performed with unpaired Student's *t*-test, and, to compare quantitative data in three groups, a one-way ANOVA and post hoc Tukey test were used.

Data from serum concentrations of adipokines, the laboratorial assessment, and disease variables were subjected to Pearson or Spearman correlation tests. The test for Hardy-Weinberg equilibrium for individual* loci* was performed (http://ihg.gsf.de/cgi-bin/hw/hwa1.pl), and contingency tables (2 × 2, 2 × 3, and 3 × 3) with *χ* ^2^ trend test or Fisher exact test, as appropriate, were used for testing the differences of genotype and phenotype distributions and allele frequencies between all subgroups.

Two genetic models were used for these analyses: (i) the dominant model where each SNP was modeled categorically and separated into three categories, one for each genotype, and (ii) the phenotype model, where each SNP was modeled into two categories, with two genotypes combined into one category (polymorphic homozygotes plus heterozygotes), choosing one genotype (homozygotes wild type) as the reference group.

Genotype intergroup comparisons by means of all variables were performed by using Student's *t*-test, one-way ANOVA for normally distributed traits, and analysis of ranks for traits with nonnormal distributions with Kruskal-Wallis or Mann-Whitney *U* tests, as appropriate. A two-tailed *P* value less than 0.05 was considered statistically significant.

## 3. Results

Clinical, demographic, and anthropometrics characteristics of the participants are shown in Table 1 split by gender because body fat mass distribution is dependent on sexual hormones. In this study overweight and obese individuals were older than those in normal range; as expected, the indicators of body fat mass* status* were different according to the classification by BMI (Table 1).

**Figure 1:**
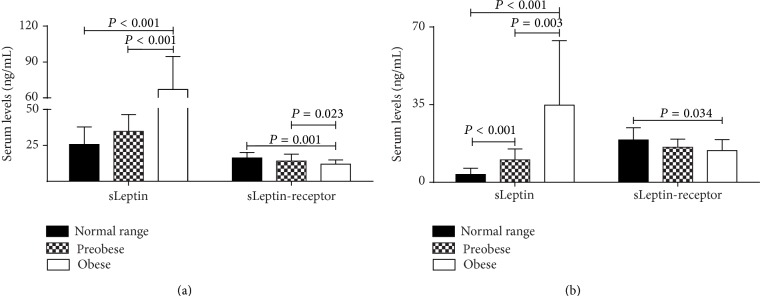
Inflammation markers, sLeptin, and sLeptin-receptor concentrations in women and men. (a) Women; (b) men. One-way ANOVA with Tukey's test; ng/mL: nanograms per milliliter.

Intergroup comparisons, according to BMI categories, both men and women with normal range versus overweight and obese, displayed higher levels of sLeptin and lower sLeptin-receptor, in BMI ≥ 25 kg/m^2^ subjects with respect to normal range individuals (Figure 1). Additionally, we observed that all the inflammation markers evaluated in this study (WBC, ESR, and high-sensitivity CRP) were higher (*P* from <0.001 to 0.039) in overweight and obese individuals with respect to those in normal range (Table 1).

A negative correlation between levels of sLeptin-receptor and body fat mass* status*, inflammation markers, and glucose was found; in an opposite manner, levels of sLeptin and CRP correlate positively (Table 2). Body fat mass determined by bioelectrical impedance analysis showed a high correlation (86%, *P* < 0.001) with skinfold body fat mass; because of this, we decided to use the first one for analyses purposes. We performed an ANCOVA where we found that body fat mass and levels of sLeptin-receptor predict 83% of the variation in the levels of sLeptin [sLeptin = −49.478 + (3.115 × body fat mass) − (1.353 × sLeptin-receptor)]. On the other hand, 76% of sLeptin-receptor levels are explained by the following equation: sLeptin-receptor = 27.66 − (0.085 × sLeptin) + (1.359 × decade of life) − (0.294 × BMI).

For both polymorphisms (*LEP* G-2548A and* LEPR* Gln223Arg), using the test for deviation from Hardy-Weinberg equilibrium, it was found that the segregation of the alleles in this Mexican Mestizo group was independent (*P* > 0.05). The analysis for* LEPR* polymorphism, using chi-square, showed association between BMI ≥ 25 kg/m^2^ and obesity with genotype and phenotype frequencies (Table 3).

**Figure 2:**
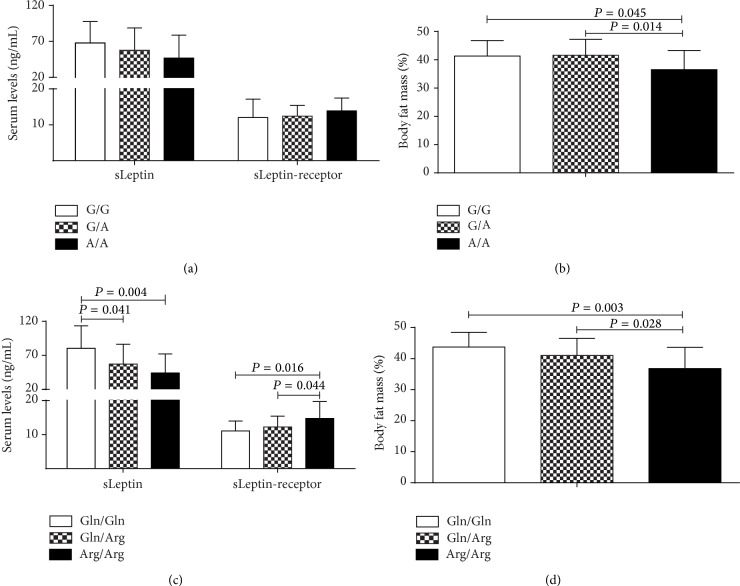
Body fat mass, sLeptin, and sLeptin-receptor in obese subjects by genotype. ((a) and (b))* LEP* G-2548A; ((c) and (d))* LEPR* Gln223Arg. One-way ANOVA with Tukey's test; ng/mL: nanograms per milliliter.

In the obese subjects we observed that Arg/Arg genotype carriers have higher levels of sLeptin-receptor and lower levels of sLeptin than Gln/Arg and Gln/Gln genotypes carriers, whereas body fat mass was lower for the A/A or Arg/Arg genotype carriers than G/G and G/A or Gln/Gln and Gln/Arg genotype carriers (Figure 2). A similar pattern in body fat mass was observed among all individuals not influenced by gender (data not shown), but statistical significance was achieved only in obese individuals.

**Figure 3:**
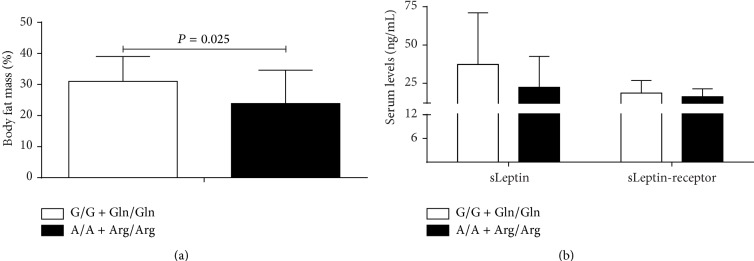
Body fat mass, sLeptin, and sLeptin-receptor, in homozygous carriers among study group. (a) Body fat mass; (b) levels of sLeptin and sLeptin-receptor. Mann-Whitney *U* test; ng/mL: nanograms per milliliter.

Additionally, among all individuals, we observed that homozygous wild genotypes double carriers (*n* = 35) showed increase in body fat mass with respect to double carriers of the homozygous polymorphic genotype (*n* = 17) (Figure 3); this analysis was not influenced by gender (data not shown). Finally, we analyzed inflammation markers in the same individuals and we found an association of polymorphic alleles with CRP serum levels (G/G + Gln/Gln = 3.4 ± 6.72 mg/dL; A/A + Arg/Arg = 1.6 ± 3.02 mg/dL).

## 4. Discussion

In this cross-sectional study 21.5% and 59.6% of the individuals were classified in the obese and overweight category, respectively, according to the criteria of the WHO. In this context, analyzing the high prevalence of overweight and obesity reported for Mexican population from the epidemiological reports perspective, it is clear that other factors besides diet and physical activity are associated with obesity development. Common obesity is considered to be polygenic but is also important to carry out studies to characterize the genetic contribution to the development of different obesity phenotypes and immune-metabolic disorders associated with overweight [2, 13, 30].

Taking into account the fact that the hallmark of obesity is the expansion and pathological accumulation of white adipose tissue, we quantified the amount and distribution of body fat mass, finding differences between individuals with normal range, overweight, and obesity for body fat mass status indicators, which not only validate our classification, but also suggest that the accumulation of white adipose tissue is progressive and the anatomical distribution differs in the states of overweight and obesity.

In previous reports, a pathogenic process has been consistently identified, defined as subclinical chronic low-grade inflammation, which can be evaluated with the profile of acute phase reactants that include ESR, WBC, and CRP. In our study group, it was observed that the levels of WBC, ESR, and CRP were higher in obese individuals than in those with normal range. These data are consistent with previous studies that characterized the presence of a proinflammatory state in obesity [31, 32], which is important because it has been seen that inflammatory process is the precursor of obesity comorbidities, among which insulin resistance, T2DM, and cardiovascular diseases are the most relevant.

Additionally, sLeptin concentrations correlated with these inflammation markers and with body fat mass accumulation; because of this, sLeptin was higher in overweight individuals with respect to normal range persons and also higher in women than in men. This is consistent with previous reports published since Rönnemaa et al. showed, in a group of homozygous twins, that leptin concentrations were higher in obese individuals compared to the nonobese, regardless of their genetic load, and higher in women than in men with similar BMI [33].

These findings are explained because leptin production is proportional to the amount of adipose tissue; so, in subjects with normal weight, increasing leptin levels suppresses the need to eat by inhibiting orexigenic neuropeptides release (e.g., neuropeptide Y and Agouti-related protein) in the hypothalamus, while obese individuals do not have this physiological response, a state called “leptin resistance.”

It has been proposed that the establishment of this condition is a consequence of the combination of three main mechanisms: diminished intracellular leptin-receptor signaling, abnormal transport of leptin across the blood-brain barrier, and development programming disorders. However, the molecular mechanisms by which lesser sensitivity to leptin is present in obesity have not yet been defined [33, 34].

Furthermore, in this study we found decreased concentrations of sLeptin-receptor in individuals with overweight and obesity, compared with individuals with normal weight, and higher concentrations in men with respect to women. Previous reports indicate that sLeptin-receptor depends on sLeptin, gender, endocrine system, and adiposity [34–36], as corroborated with ANCOVA test.

Summarizing, it was determined that sLeptin correlated positively with BMI and body fat mass percentage; conversely, sLeptin-receptor showed a negative correlation. This inverse relationship is consistently reported in previous studies [34–36]. Besides, it has been described that sLeptin-receptor has at least two functions: (i) retarding leptin degradation and therefore helping to maintain serum concentrations and (ii) suppressing leptin action by preventing its binding to membrane receptors, which is associated with less transport of leptin across blood-brain barrier.

The adjustment mechanism whereby leptin may modulate levels of sLeptin-receptor or inversely has not been established; nonetheless, there are some studies in animal models [35, 37] and humans [34] that give us a clue. A diminution of sLeptin-receptor concentration is detected hours after recombinant leptin administration in mice; also, a decrease of sLeptin-receptor occurs in critically ill patients who have increased levels of leptin. These results suggest that regulation may occur at a posttranslational level [35].

In the context of genetic framework for* LEP* and* LEPR* in our Mexican Mestizo group, the distribution of genotypes for* LEP* G-2548A polymorphism showed no difference between individuals with normal range, overweight, and obesity; however, we performed a detailed analysis that will be described later.

On the other hand, for* LEPR* Gln223Arg polymorphism, we found differences in the proportions of genotypes and phenotypes within overweight and obese individuals, compared to normal range. In this analysis we observed higher heterozygous genotype and Arg+ phenotype frequencies in BMI ≥ 25 kg/m^2^ subjects; this finding suggests that Gln223Arg polymorphism may be related to obesity in Western Mexican Mestizo population (Table 3).

The association of these polymorphisms with the development of obesity and comorbidities reported in different and previous studies found conflicting results; for example, in populations of Taiwan [38] and Brazil [39], the distribution of genotypes is different from that found in this group of Mexican Mestizos, while Romanians [40] and Polish [17] populations are similar.

In other reports about* LEP* G-2548A polymorphism in Spanish teenagers [41] and* LEPR* Gln223Arg polymorphism in teenagers from Guanajuato, Mexico [42], the distributions of the genotypes were different with respect to our study group. These data suggest that the distribution of genotypes in different populations is concerned with its anthropological background, findings that highlight the importance of genetic characterization of populations groups.

Despite of that, association of* LEP* G-2548A and* LEPR* Gln223Arg polymorphisms with obesity and leptin serum levels in other populations has been studied with controversial results [18, 43, 44], and the relationship between leptin and leptin-receptor polymorphisms with the serum levels of both molecules (sLeptin and sLeptin-receptor) and adiposity has not been explored.

Due to difference in the body fat mass percentage found among obese individuals carriers of different genotypes for both polymorphisms (Figure 2), we analyzed adiposity among all subjects carrying both homozygous polymorphic genotypes (-2548A/A at* LEP* and 223Arg/Arg at* LEPR*), and we found association with lower percentage of body fat mass on them (Figure 3). In parallel, sLeptin and sLeptin-receptor levels in obese individuals showed a trend with respect to* LEP* G-2548A genotypes, while, for* LEPR* 223Arg/Arg, the levels of sLeptin and sLeptin-receptor levels were lower and higher, respectively.

This partially agrees with reports by Riestra, Constantin, and Hinuy associating higher levels of sLeptin with genotype -2548G/G [39–41]. Yiannakouris et al. reported increased levels of sLeptin-receptor in -2548A/A within Greek young women [45], and Guízar-Mendoza et al. showed association of Gln223 allele with higher levels of sLeptin [42]; however, there are no previous studies where sLeptin-receptor levels are quantified together with the presence of* LEPR* Gln223Arg polymorphism.

Other reports have shown association of allele -2548G with obesity in Spaniards [41], Brazilians [39], and Romanian population [40] and in individuals with obesity types II and III (BMI > 35 kg/m^2^) [38], while, for 223Arg/Arg genotype, a relation with lower BMI and fat percentage is reported [42]. These data suggest that the degree of association shows a dependence on ethnicity background and gender.

Among the most relevant data for this study we found two scenarios: (1) we observed in obese individuals carriers of wild type alleles (-2548G of* LEP *and Gln223 of* LEPR*) increased sLeptin levels and adiposity but lower levels of sLeptin-receptor; (2) conversely, in obese individuals carriers of polymorphic alleles (-2548A of* LEP *and 223Arg of* LEPR*), a reduction in sLeptin levels and adiposity was seen, but higher sLeptin-receptor serum concentration was seen. With respect to this, Fuentes et al. [46] observed that a selective state of leptin resistance can be observed in skeletal muscle from obese patients. In a similar way SNPs in* LEP* and* LEPR* could affect the expression of leptin-receptor and leptin resistance in skeletal muscle.

This can be explained based on two assumptions:

1. The allele -2548G improves the transcription rate of* LEP*, which is returned in increased basal levels of leptin, something that can, early and indirectly, affect the expression of leptin-receptor in membrane and serum levels. This process maintains fat accumulation due to decreased anorexigenic signaling, a phenomenon that generates the state of “leptin resistance.”
2. Even though the 223Arg allele of* LEPR* encodes a protein with leptin signaling altered capacity, the levels of soluble leptin-receptor are increased and a delay in the establishment of leptin resistance is promoted if the cells expressing the leptin-receptor decrease the leptin signaling; therefore, body fat mass accumulation is lower and orexigenic signaling remains active.

## 5. Limitations

Differences in physical activity (PA) among individuals could be important since it has been observed that short-term physical training may result in modification of sLeptin-receptor [47]. Unfortunately we did not evaluate the level of PA in study subjects; hence, we were not able to include a PA in multivariate analysis.

## 6. Conclusions

Our results suggest that levels of sLeptin and sLeptin-receptor reflect the state of adiposity in obese individuals because they are associated with BMI, inflammatory markers, and body fat mass distribution; and the polymorphic alleles* LEP* -2548A and* LEPR* 223Arg could be genetic markers associated with less body fat mass accumulation in obese subjects from Western Mexico population.

## Figures and Tables

**Table 1:** Demographic, clinical, and anthropometrics measures in study group according to WHO classification.

	Female				Male			
	Normal range	Overweight	Obese	*P*	Normal range	Overweight	Obese	*P*
	(BMI 18.5–24.9 kg/m^2^)	(BMI 25.0–29.9 kg/m^2^)	(BMI ≥ 30 kg/m^2^)		(BMI 18.5–24.9 kg/m^2^)	(BMI 25.0–29.9 kg/m^2^)	(BMI ≥ 30 kg/m^2^)	
	*n* = 104	*n* = 86	*n* = 60		*n* = 50	*n* = 60	*n* = 22	
Age (years)	33.4 ± 13.72	42.9 ± 12.45	42.8 ± 11.77	<0.001^∗^	30.3 ± 12.95	39.6 ± 12.84	41.4 ± 10.10	<0.004^∗^
Blood pressure (mmHg)								
Diastolic	70 ± 7.6	75 ± 7.3	79 ± 9.4	<0.001^∗^	72 ± 9.0	75 ± 8.6	79 ± 9.4	0.020^#^
Systolic	106 ± 9.4	118 ± 13.1	120 ± 17.9	<0.001^∗^	115 ± 10.1	117 ± 11.9	122 ± 12.8	NS
Hemoglobin (g/dL)	13.9 ± 1.06	13.6 ± 1.21	14.1 ± 1.00	0.027^+^	16.3 ± 1.15	16.1 ± 1.08	15 ± 1.1	NS
Glucose (mg/dL)	87 ± 13.0	96 ± 11.8	96 ± 15.6	<0.001^∗^	87 ± 10.7	96 ± 15.0	103 ± 15.3	<0.001^∗^
White blood cells (k/*µ*L)	5.8 ± 1.44	6.1 ± 1.29	6.5 ± 1.54	0.009^#^	5.8 ± 1.91	6.3 ± 1.27	7.3 ± 2.04	0.015^#^
Erythrocyte sedimentation rate (mm/h)	17 ± 9.2	23 ± 9.4	24 ± 9.9	<0.001^∗^	5 ± 5.8	9 ± 7.5	11 ± 7.0	0.025^∗^
C reactive protein (mg/dL)	1.4 ± 1.93	3.9 ± 3.22	4.3 ± 3.30	<0.001^∗^	1.1 ± 1.61	2.7 ± 2.59	4.5 ± 4.07	<0.039^∗^
Height (cm)	158.9 ± 14.88	157.6 ± 5.95	159.2 ± 8.16	—	173.4 ± 6.07	171.5 ± 6.09	172.5 ± 8.28	—
Weight (kg)	57.8 ± 7.36	68.0 ± 6.14	86.2 ± 12.34	—	67.7 ± 7.19	80.9 ± 7.52	101.2 ± 12.83	—
Body mass index (kg/m^2^)	22.4 ± 1.61	27.3 ± 1.41	33.9 ± 3.63	—	22.4 ± 1.78	27.4 ± 1.45	34.0 ± 4.21	—
Waist circumference (cm)	76.2 ± 7.61	88.7 ± 6.75	102.7 ± 10.30	<0.001	82.5 ± 7.71	95.8 ± 8.01	110.9 ± 10.55	<0.001
Hip circumference (cm)	96.2 ± 5.89	103.5 ± 4.99	117.4 ± 9.49	<0.001	94.1 ± 6.33	101.3 ± 5.15	113.1 ± 11.15	<0.001
Waist/hip ratio	0.790 ± 0.0604	0.856 ± 0.0647	0.881 ± 0.0843	<0.001^∗^	0.871 ± 0.0880	0.946 ± 0.0722	0.990 ± 0.0864	<0.001^∗^
Body fat mass (%)	27.2 ± 5.53	36.1 ± 4.10	43.1 ± 3.40	<0.001	15.0 ± 5.18	25.8 ± 4.00	33.6 ± 6.47	<0.001
Skinfold thickness (mm)								
Biceps	11.6 ± 4.70	17.4 ± 5.34	23.5 ± 8.16	<0.001	5.2 ± 2.58	9.6 ± 5.11	16.4 ± 7.46	<0.022
Triceps	20.6 ± 4.67	27.4 ± 5.28	33.6 ± 8.57	<0.001	11.7 ± 5.23	16.4 ± 5.94	22.1 ± 7.96	<0.012
Subscapular	20.4 ± 7.90	30.2 ± 6.98	38.8 ± 8.57	<0.001	15.1 ± 6.48	25.3 ± 7.12	37.3 ± 11.18	<0.001
Suprailiac	23.2 ± 6.46	29.9 ± 5.98	35.0 ± 7.74	<0.002	17.4 ± 8.07	26.3 ± 8.43	35.1 ± 9.78	<0.006

mmHg: millimeter of mercury; g: gram; dL: deciliter; kg: kilogram; cm: centimeter; mm: millimeter; m^2^: square meter.  ^∗^Normal range versus overweight and obese;  ^+^overweight versus obese;  ^#^normal range versus obese; k: thousand; *μ*L: microlitre.

**Table 2:** sLeptin and sLeptin-receptor correlations in the study group.

	sLeptin	sLeptin-receptor	CRP
	Correlation coefficient (%, *P*)		
Waist circumference (cm)	**40**	−**22**	**38**
	**<0.001**	**0.012**	**<0.001**

Hip circumference (cm)	**65**	**−43**	**40**
	**<0.001**	**<0.001**	**<0.001**

Waist/hip ratio	−5	8	**15**
	0.510	0.329	**0.030**

Body mass index (kg/m^2^)	**66**	**−44**	**44**
	**<0.001**	**<0.001**	**<0.001**

Body fat mass (%)	**77**	**−49**	**45**
	**<0.001**	**<0.001**	**<0.001**

Glucose (mg/dL)	−1	6	**36**
	0.909	0.479	**<0.001**

White blood cells (k/*μ*L)	**22**	**−30**	**37**
	**0.006**	**<0.001**	**<0.001**

Erythrocyte sedimentation rate (mm/h)	**48**	**−20**	**37**
	**<0.001**	**0.011**	**<0.001**

C reactive protein (mg/L)	**37**	**−24**	—
	**0.001**	**0.041**	

sLeptin-receptor	**−55**	—	**−24**
	**<0.001**		**0.041**

*n* = 382, Pearson correlationstest, m^2^: square meter; cm: centimeter; L: liter; dL: deciliter; *μ*L: microliter; kg: kilogram; mg: milligram; ng: nanogram; h: hour; %: percentage.

**Table 3:** Distribution of *LEP* G-2548A and *LEPR* Gln223Arg polymorphisms in Mexican Mestizo population.

Study group	Genotype *n* (%)			Phenotype *n* (%)	Allele *n* (%)	
*LEP* G-2548A	G/G	G/A	A/A	A+	G	A

Normal range (BMI 18.5–24.9 kg/m^2^)	48 (31.2)	78 (50.6)	28 (18.2)	106 (68.8)	174 (56)	134 (44)
Overweight (BMI 25.0–29.9 kg/m^2^)	52 (35.6)	72 (49.3)	22 (15.1)	94 (64.4)	176 (60)	116 (40)
Obese (BMI ≥ 30 kg/m^2^)	21 (25.6)	47 (57.3)	14 (17.1)	61 (74.4)	89 (54)	75 (46)
*P*	NS			NS	NS	
BMI ≥ 25 kg/m^2^	73 (32)	119 (52.2)	36 (15.8)	155 (68)	265 (58)	191 (42)
*P*	NS			NS	NS	

*LEPR* Gln223Arg	Gln/Gln	Gln/Arg	Arg/Arg	Arg+	Gln	Arg

Normal range (BMI 18.5–24.9 kg/m^2^)	52 (33.8)	65 (42.2)	37 (24)	102 (66.2)	169 (55)	139 (45)
Overweight (BMI 25–29.9 kg/m^2^)	37 (25.3)	84 (57.6)	25 (17.1)	109 (74.7)	158 (54)	134 (46)
Obese (BMI ≥ 30 kg/m^2^)	15 (18.3)	50 (61)	17 (20.7)	67 (81.7)	80 (49)	84 (51)
*P *	0.0197^*α*^			0.0319^*β*^	NS	
BMI ≥ 25 kg/m^2^	52 (22.8)	134 (58.8)	42 (18.4)	176 (77.2)	238 (52)	218 (48)
*P*	0.0058^*γ*^			0.0196^*δ*^	NS	

*n* = 382; NS: nonsignificance; A+: G/A plus A/A; A−: G/G; Arg+: Gln/Arg plus Arg/Arg; Arg−: Gln/Gln; categorical variables were analyzed using *χ* ^2^ or Fisher exact test, accordingly. ^*α*^Normal range, overweight, and obese by genotype; ^*β*^normal range, overweight, and obese by phenotype; ^*γ*^normal range and BMI ≥ 25 kg/m^2^ by genotype; ^*δ*^normal range and BMI ≥ 25 kg/m^2^ by phenotype.
